# Immunohistochemical Detection of Potential Microbial Antigens in Granulomas in the Diagnosis of Sarcoidosis

**DOI:** 10.3390/jcm10050983

**Published:** 2021-03-02

**Authors:** Tetsuo Yamaguchi, Ulrich Costabel, Andrew McDowell, Josune Guzman, Keisuke Uchida, Kenichi Ohashi, Yoshinobu Eishi

**Affiliations:** 1 Department of Human Pathology, Graduate School and Faculty of Medicine, Tokyo Medical and Dental University, Tokyo 113-8519, Japan; yamatet@icloud.com (T.Y.); uchida.path@tmd.ac.jp (K.U.); kohashi.pth1@tmd.ac.jp (K.O.); 2 Department of Pulmonology, Shinjuku Tsurukame Clinic, Tokyo 151-0053, Japan; 3 Department of Pneumology, Ruhrlandklinik, Medical Faculty, University of Duisburg-Essen, 45239 Essen, Germany; Ulrich.Costabel@rlk.uk-essen.de; 4 Nutrition Innovation Centre for Food and Health (NICHE), School of Biomedical Sciences, Ulster University, Coleraine BT52 1SA, UK; a.mcdowell@ulster.ac.uk; 5 Department of General and Experimental Pathology, Ruhr University, 44801 Bochum, Germany; josune.guzman@rub.de

**Keywords:** pathogenesis, quantitative PCR, hypersensitivity, immunohistochemistry, symbiosis, endogenous infection, *Propionibacterium acnes*, *Corynebacterium parvum*, *Cutibacterium acnes*, *Mycobacterium tuberculosis*

## Abstract

Sarcoidosis may have more than a single causative agent, including infectious and non-infectious agents. Among the potential infectious causes of sarcoidosis, *Mycobacterium tuberculosis* and *Propionibacterium acnes* are the most likely microorganisms. Potential latent infection by both microorganisms complicates the findings of molecular and immunologic studies. Immune responses to potential infectious agents of sarcoidosis should be considered together with the microorganisms detected in sarcoid granulomas, because immunologic reactivities to infectious agents reflect current and past infection, including latent infection unrelated to the cause of the granuloma formation. Histopathologic data more readily support *P. acnes* as a cause of sarcoidosis compared with *M. tuberculosis*, suggesting that normally symbiotic *P. acnes* leads to granuloma formation in some predisposed individuals with Th1 hypersensitivity against intracellular proliferation of latent *P. acnes*, which may be triggered by certain host or drug-induced conditions. Detection of bacterial nucleic acids in granulomas does not necessarily indicate co-localization of the bacterial proteins in the granulomas. In the histopathologic diagnosis of sarcoidosis, *M. tuberculosis*-associated and *P. acnes*-associated sarcoidosis will possibly be differentiated in some patients by immunohistochemistry with appropriate antibodies that specifically react with mycobacterial and propionibacterial antigens, respectively, for each etiology-based diagnosis and potential antimicrobial intervention against sarcoidosis.

## 1. Introduction

Sarcoidosis is a systemic inflammatory disease that is characterized by the formation of noncaseating epithelioid cell granulomas at the sites of disease activity in multiple organs, including the lungs and lymph nodes [1]. Despite numerous studies using molecular and immunologic approaches, the cause of sarcoidosis remains uncertain. Sarcoidosis may have more than a single causative agent, including infectious and non-infectious agents [2]. Even if a specific microorganism is involved, the infectious agent does not need to cause sarcoidosis in every host or experimental animal according to Koch’s postulates for establishing causation of an infectious disease [3].

Among the potential infectious agents, *Mycobacterium tuberculosis* and *Propionibacterium acnes* (formerly known as *Corynebacterium parvum* and currently referred to as *Cutibacterium acnes*) [4] are the most likely causative microorganisms of sarcoidosis. Which of the two infectious agents is more likely to contribute to the pathogenesis among sarcoidosis patients worldwide, however, remains uncertain. This paper reviews the evidence supporting mycobacterial and propionibacterial etiologies of sarcoidosis, and describes a potential pathogenesis based on molecular, immunologic, and histopathologic investigations.

## 2. Causative Agents of Granuloma Formation

Granulomas serve as a protective mechanism to confine poorly degradable extrinsic agents [5]. Foreign body granulomas are formed by agents with weak antigenicity (e.g., surgical sutures), whereas epithelioid cell granulomas are formed by agents with strong antigenicity that can induce an active Th1 immune response [6]. In infectious diseases, microorganisms usually act as both foreign bodies and antigens that induce immunologic responses [7].

Histologically, granulomas start to form as small aggregations of lymphocytes and macrophages around poorly degraded antigens. At the beginning of granuloma formation, macrophages change to epithelioid cells and organize into cell clusters (immature granuloma). As the granuloma progresses, a ball-like cluster of epithelioid cells develops, which is occasionally accompanied by the fusion of macrophages into giant cells (mature granuloma). In granulomas caused by infectious or non-infectious agents, the causative agent is present or has been present in the granulomas [5]. To identify a certain agent as the cause of Th1 granuloma formation, evidence of its presence in the granulomas as well as antigenic hypersensitivity in the patient must be established. This concept of infectious granuloma pathogenesis is the same as that in cases of non-infectious granulomas, such as berylliosis, and must be considered when searching for unknown causative agents of sarcoidosis.

While histopathologic investigations are useful for detecting and locating the causative agents in granulomas, the extrinsic agents or antigens in the granulomas are usually degraded or abolished by the granuloma cells, which have a greater intracellular digestive ability than conventional macrophages [8,9]. Therefore, the causative agent in granulomas may no longer be present, and may only be observed in a few, if any, tissue sections of the granulomas. Because of the degradation process in the granuloma, the causative agent is more likely to be identified in immature granulomas with many inflammatory cells than in mature granulomas with only a few lymphocytes. When a microorganism is detected in granulomas, it is highly suspected as the cause, but even when no microorganism is identified, a microbial etiology cannot be excluded. In infectious granulomas, microbial antigens detected by immunohistochemistry (IHC) are more likely to be degraded or abolished compared with microbial DNA detected by polymerase chain reaction (PCR) or in situ hybridization methods.

## 3. Microorganisms Detected in Sarcoid Tissues

Due to the common features of sarcoidosis and tuberculosis, a mycobacterial cause of sarcoidosis has been suggested since the first description of the disease over a century ago. Although mycobacteria have not been found in sarcoid tissues by conventional histologic and culture techniques [10], a mycobacterial etiology was hypothesized after successful PCR detection of *M. tuberculosis* DNA in sarcoid tissues [11], including granulomas and tissues outside the granulomas. On the basis of a meta-analysis [12] of 31 studies using qualitative PCR published from 1980 to 2006, the odds ratio (OR) for identifying mycobacterial DNA, including *M. tuberculosis* complex (MTC) and nontuberculous mycobacteria (NTM), in sarcoidosis versus control samples was calculated to be 9.67; mycobacterial DNA was detected in 231 (26%) of 874 sarcoidosis samples (MTC: 187, NTM: 43, and both: 1) and in 17 (3%) of 600 control samples (MTC: 13, NTM: 2, and unknown: 2). The detection frequencies in sarcoid tissues were greater than 50% in seven studies, 20% to 50% in eight studies, less than 20% in nine studies, and 0% in seven studies.

**Figure 1:**
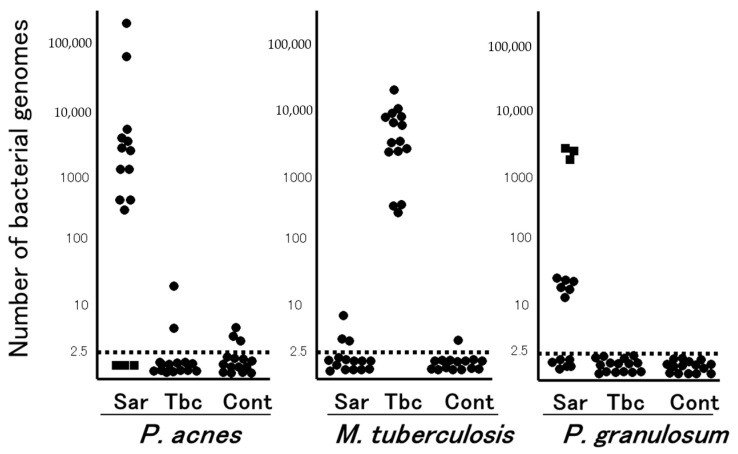
Quantitative PCR for mycobacterial and propionibacterial DNA in sarcoid lymph nodes. Lymph node samples from each of the 15 patients with sarcoidosis (Sar), tuberculosis (Tbc), and gastric cancer (Cont) were used in this study. The horizontal dotted lines show the detection threshold and samples with results below this line are considered negative. Samples without *P. acnes* detected (as indicated by dotted squares) all contained many *P. granulosum* (reproduced from Ishige et al. [15] with permission from the Lancet, London).

In the late 1970s, the Japanese government supported extensive efforts to isolate microorganisms such as bacteria, viruses, and fungi from sarcoid tissues, which unexpectedly led to the isolation of only *P. acnes* and no other microorganism, including mycobacteria, from sarcoid tissues [13]; *P. acnes* was isolated from 78% of 40 sarcoidosis and 21% of 180 control lymph nodes [14]. Quantitative PCR led to the detection of many *P. acnes* genomes in 80% of sarcoidosis samples and only a few *P. acnes* genomes in 17% of non-sarcoidosis samples (Figure 1). Many *M. tuberculosis* genomes were detected in all tuberculosis samples and a few were detected in 13% of non-tuberculosis samples [15]. Consequently, an international collaborative study on lymph node samples was performed in Japan, Italy, Germany, and England using quantitative real-time PCR [16]; either *P. acnes* or *P. granulosum* was detected in all but two sarcoidosis samples. *M. tuberculosis* was detected in 0% to 9% of sarcoidosis samples and 65% to 100% of tuberculosis samples. In sarcoid lymph node samples from each country, the observations of propionibacterial genomes far outnumbered those of *M. tuberculosis* genomes.

The results of a recent meta-analysis of 58 studies involving more than 6000 patients in several countries evaluating all types of infectious agents proposed to be associated with sarcoidosis suggested an etiologic link with *P. acnes* (OR: 18.8, 95%; CI: 12.6, 28.1) and mycobacteria (OR: 6.8, 95%; CI: 3.7, 12.4) [17]. Other infectious agents such as *Borrelia* (OR: 4.8), HHV-8 (OR: 1.5) as well as *Rickettsia helvetica*, *Chlamydia pneumoniae*, Epstein–Barr virus, and retrovirus, although suggested by previous investigation, were not associated with sarcoidosis.

Bacterial DNA was detected in lymph nodes from 11 (37%) of 30 sarcoidosis patients (*P. acnes*: 6, NTM: 3, and others: 2) and 2 (7%) of 30 control patients (*P. acnes*: 1 and NTM: 1) using 16S-rRNA gene sequencing [18]. Similarly, high-throughput 16S-rRNA gene sequencing revealed a significantly higher relative abundance of *P. acnes* in lymph node samples obtained from a sarcoidosis group (0.16%) than in those obtained from control (0%) and tuberculosis (0%) cohorts, while the relative abundance of mycobacterium did not differ between sarcoidosis (0.06%) and control (0.05%) samples [19].

## 4. Latent Infection and Endogenous Reactivation

Primary *M. tuberculosis* infection, most often occurring in childhood, leads to disease in only about 10% of those infected. Some bacilli in a latent state may remain in the tissues throughout the individual’s life and most cases of tuberculosis (secondary tuberculosis) are due to endogenous reactivation of latent infection. *M. tuberculosis* components are abundant in old tuberculosis lesions with calcification [20] and may persist intracellularly in alveolar and interstitial macrophages, type II pneumocytes, endothelial cells, and fibroblasts in lung tissue without histologic evidence of tuberculosis lesions [21].

*P. acnes* is a gram-positive anaerobic bacterium that is part of the normal microbiota of the skin, oral cavity, and gastrointestinal and genitourinary tracts [22]. *P. acnes* survives intracellularly and persists in macrophages without intracellular replication [23]. An overload of *P. acnes* in macrophages induces autophagy and some *P. acnes* remain persistent in the macrophages [24]. Intracellular *P. acnes* has been identified in alveolar and sinus macrophages in the lungs and lymph nodes, respectively [25]. Some colonies of *P. acnes* can be isolated from peripheral lungs and mediastinal lymph nodes, even in the absence of inflammation [26]. *P. acnes* can invade epithelial cells [27,28] and some persist intracellularly [29].

Potential latent infection of *M. tuberculosis* and *P. acnes* in the lungs and lymph nodes implies that a few bacterial genomes detected by PCR reflect latent infection unrelated to the disease. Quantitative PCR may help to discriminate between a latent and reactivated status of each bacterium detected in tissues with granulomatous inflammation. Zhou et al. [30] used quantitative PCR for *M. tuberculosis* DNA to differentiate between tuberculosis and sarcoidosis lesions; the genomes were detected in 100% of tuberculosis samples, 19% of sarcoidosis samples, and 13% of control tissue samples (detection specificity for tuberculosis: 81% with an assay sensitivity of 10^2^ genome copies/mL), whereas the specificity was increased to 98% with a cut-off value of 1.14 × 10^3^ genome copies/mL. They also reported the results of quantitative reverse transcription-PCR for propionibacterial rRNA using lymph node samples [31]; *P. acnes* or *P. granulosum* rRNA was detected in 74% of 65 sarcoidosis samples, 9% of 45 tuberculosis samples, and 6% of 50 control samples. With a cut-off value of 50 genome copies/mL, the propionibacterial rRNA was positive in an additional 79% of 24 sarcoidosis samples and an additional 4% of 22 tuberculosis samples (detection specificity for sarcoidosis: 96%). Rotsinger et al. [32] used quantitative real-time PCR for multiple mycobacterial genes and reported that 1 or more of the 6 mycobacterial genes was detected in 85% of 33 sarcoidosis samples and 7% of 30 control samples, although the quantitative results were not described.

## 5. Immune Responses to *M. tuberculosis* and *P. acnes*

Fang et al. [33] conducted a meta-analysis of studies on immune responses to mycobacteria, which included nine reports on T-cell immune responses and four reports on humoral immune responses. The stimulating antigens used in most studies were from *M. tuberculosis*, including early secretory antigenic target (ESAT)-6 and mycobacterial catalase-peroxidase (mKatG). In many reports, the T-cell or humoral immune responses were significantly higher in sarcoidosis patients than in the control group. Therefore, the authors concluded that *M. tuberculosis* may be associated with the pathogenesis of sarcoidosis. According to the results of tuberculin skin tests, however, the control group included purified protein derivative (PPD)-negative, PPD-positive, and PPD-unknown cases, and the difference between the sarcoidosis and control groups was no longer significant when compared with the PPD-positive healthy control group. Sarcoidosis patients may be prone to latent *M. tuberculosis* and *P. acnes* infection due to unknown host factors including decreased expression levels of nucleotide-binding oligomerization domain-containing protein 1 (NOD1) in response to intracellular infection by these gram-positive bacteria [28].

The QuantiFERON-TB Gold test is a simple interferon-gamma (IFN-ɣ) release assay with two stimulating antigens, ESAT-6 and culture filtrate protein-10, both specific to *M. tuberculosis* infection. Several studies reported the positivity rate in sarcoidosis patients— 3% in Japan [34], 7% in Denmark [35], 21% in Poland [36], and 34% in India [37]—with no significant difference in any report between sarcoidosis patients and healthy controls in each country. In studies that simultaneously investigated the reactivities to PPD, ESAT-6, and culture filtrate protein-10, IFN-ɣ release by peripheral blood mononuclear cells and bronchoalveolar lavage (BAL) cells in response to these *M. tuberculosis* antigens did not differ significantly between patients with sarcoidosis and those with control lung diseases [38,39].

Furusawa et al. [40] reported significant differences in the Th1/Th17 responses of peripheral blood mononuclear cells between sarcoidosis and control groups when stimulated with viable *P. acnes* but not with viable MTC (bacillus Calmette–Guérin) or ESAT-6; the increased interleukin-2 and decreased interleukin-17 responses to *P. acnes* suggested an imbalance of Th1/Th17 immune responses to the commensal bacterium in sarcoidosis patients. Cellular and humoral immune responses to *P. acnes* trigger factor [41] and catalase [42] are increased in Japanese sarcoidosis patients. German patients with sarcoidosis also had high levels of specific antibodies to *P. acnes* in the BAL fluid, and the BAL cells in those patients produced inflammatory cytokines (tumor necrosis factor alpha and granulocyte-macrophage colony-stimulating factor) upon stimulation with heat-killed *P. acnes* [43]. IFN-ɣ release from BAL cells upon stimulation with the supernatant of boiled *P. acnes* did not differ between sarcoidosis and control patients in the United States [44].

Immune responses to potential infectious agents of sarcoidosis should be considered together with the microorganisms detected in sarcoid granulomas, because immunologic reactivities to infectious agents reflect current and past infection, including latent infection unrelated to the cause of the granuloma formation.

## 6. *M. tuberculosis* and *P. acnes* in Sarcoid Granulomas

Four studies investigated the localization of mycobacterial components in sarcoid tissues. *M. tuberculosis* KatG and 16S-rRNA DNA were detected in formalin-fixed paraffin-embedded (FFPE) sarcoid tissues by in situ hybridization with tyramide signal amplification [45]. Mycobacterial ESAT-6 was detected by matrix-assisted laser desorption-ionization imaging mass spectrometry at sites of granulomatous inflammation in snap-frozen sarcoid specimens [44]. Mycobacterial *gyrA* nucleic acids were localized to sites of granuloma formation by a novel in situ RNA detection kit (RNAscope) [32]. In only one report, mycobacterial antigens were located by IHC with commercially available monoclonal antibodies; *M. tuberculosis* hsp70, hsp65, and hsp16 were detected in all cases of snap-frozen sarcoid lymph nodes, while *M. tuberculosis* DNA was detected in 6% of the sarcoid tissues in the study [46]. In the four studies mentioned above, the methods and tissues used to locate *M. tuberculosis* DNA or antigens in sarcoid granulomas varied, and none of the results have been validated by others. Additionally, IHC with a commercially available LAM antibody (D372-3, MBL, Japan), which reacts with mycobacterial lipoarabinomannan in FFPE tissues, detected positive signals in tuberculosis granulomas [20], whereas no signal was detected in sarcoid granulomas (Y. Eishi, unpublished observation).

**Figure 2:**
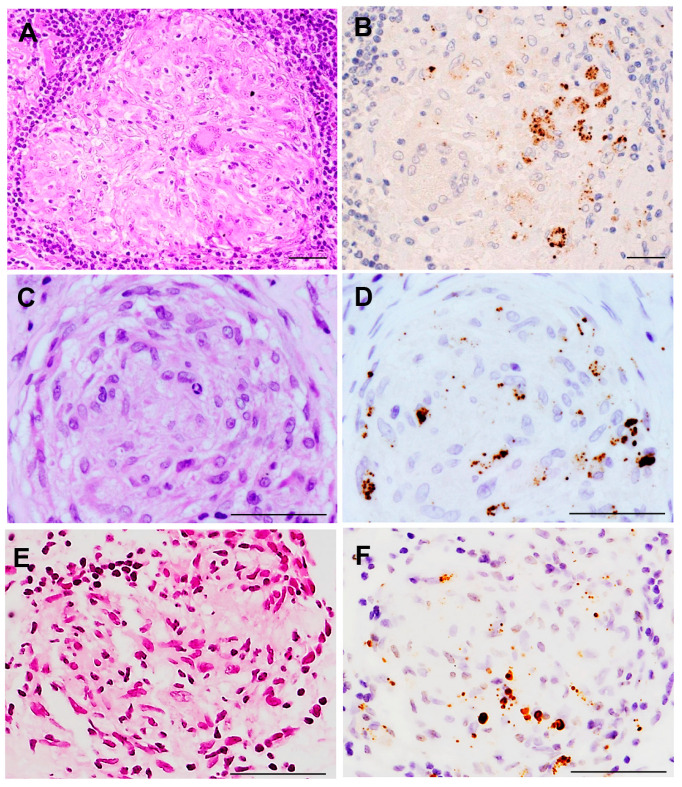
*P. acnes* in sarcoid granulomas detected by immunohistochemistry with PAB antibody. Hematoxylin–eosin stain and immunohistochemistry with *P. acnes*-specific PAB antibody are shown pairwise. PAB-reactive *P. acnes* (resulting in brown color) are observed in non-caseating epithelioid cell granulomas of the lymph node (**A**,**B**), lung (**C**,**D**), and ocular epiretinal membrane (**E**,**F**) from patients with sarcoidosis. Mainly small round and occasionally large ovoid positive-signals are observed in granuloma cells, irrespective of the sites at which the granuloma formed. All photos are original. Scale bar: 50 μm.

**Figure 3:**
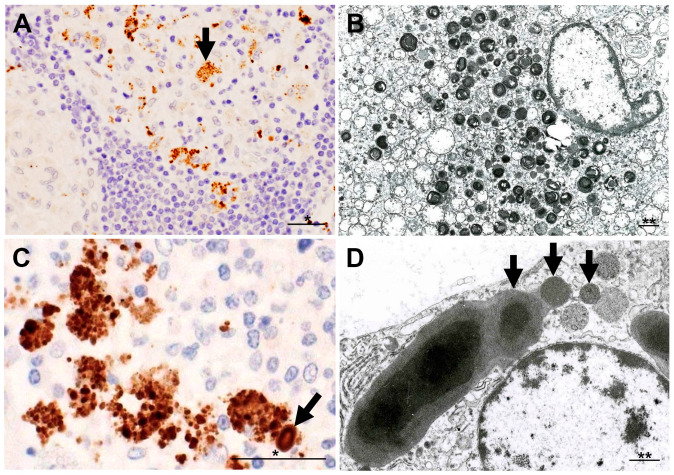
Morphologic features of intracellular *P. acnes* proliferation in sarcoid lymph nodes. Most of the PAB-reactive *P. acnes* in granuloma cells, such as indicated by an arrow (**A**), are degraded showing lamellar body formation (**B**). Intracellular proliferation of *P. acnes* is suggested in an early focus of paracortical granuloma composed of a cluster of swollen macrophages filled with many PAB-reactive small round bodies with a similarly PAB-reactive Hamazaki–Wesenberg (HW) body (an arrow) (**C**). HW bodies are found mainly in sinus macrophages rarely accompanied by small round bodies (arrows) sprouting from the large ovoid HW body (**D**). All photos are original. Scale bar: * 50 μm, ** 1 μm.

*P. acnes* 16S-rRNA DNA was detected in sarcoid granulomas of FFPE lymph nodes by in situ hybridization with signal amplification by catalyzed reporter deposition [47]. This method was also used to detect *P. acnes* DNA in granulomas of necrotizing sarcoid granulomatosis [48]. The commercially available PAB antibody (D371-3, MBL) is a *P. acnes*-specific monoclonal antibody that reacts with cell membrane-bound lipoteichoic acid of the bacterium in FFPE tissues. IHC with the PAB antibody has detected *P. acnes* in sarcoid granulomas of various organs (Figure 2). Positive signals in sarcoid granulomas were found in 88% of 81 Japanese and 89% of 38 German lymph node samples, whereas no signal was detected in non-sarcoidosis granulomas, such as tuberculosis and sarcoid reaction [25]. Histopathologic analysis suggested latent infection and intracellular proliferation of *P. acnes* in sarcoid lymph nodes (Figure 3) [25,49,50]. The frequent identification of *P. acnes* in sarcoid granulomas of originally aseptic organs, such as the heart [51] and eyeball [52,53], provides further evidence linking this commensal bacterium to the cause of granuloma formation. Many case reports with *P. acnes* detected in granulomas, including pulmonary sarcoidosis [54,55], cutaneous sarcoidosis [56,57,58,59,60,61,62], nasal sarcoidosis [63], and neurosarcoidosis [64,65], have been published. These cases in which *P. acnes* was identified in granulomas by IHC with the PAB antibody were recently termed *P. acnes*-associated sarcoidosis [55,64,65,66,67,68].

## 7. Potential Pathogenesis of Sarcoidosis

The absence of active mycobacterial infection suggests that only some specific, poorly degraded mycobacterial components act as antigens and contribute to granulomatous inflammation in subjects with Th1 hypersensitivity [33]. *M. tuberculosis* KatG is the most implicated tissue antigen and target of the adoptive immune response in sarcoidosis [69]. Recent studies proposed a role for serum amyloid A in promoting a progressive and chronic granulomatous inflammation in the absence of ongoing infection [70], and even a role for vimentin as a possible autoantigen in sarcoidosis patients [71]. It is important, however, to demonstrate the causative agents in sarcoid granulomas. Detection of mycobacterial nucleic acids in granulomas does not necessarily indicate co-localization of the bacterial proteins in the granulomas. IHC with anti-mKatG or other anti-mycobacterium antibodies may be useful for diagnosing *M. tuberculosis*-associated sarcoidosis.

**Figure 4:**
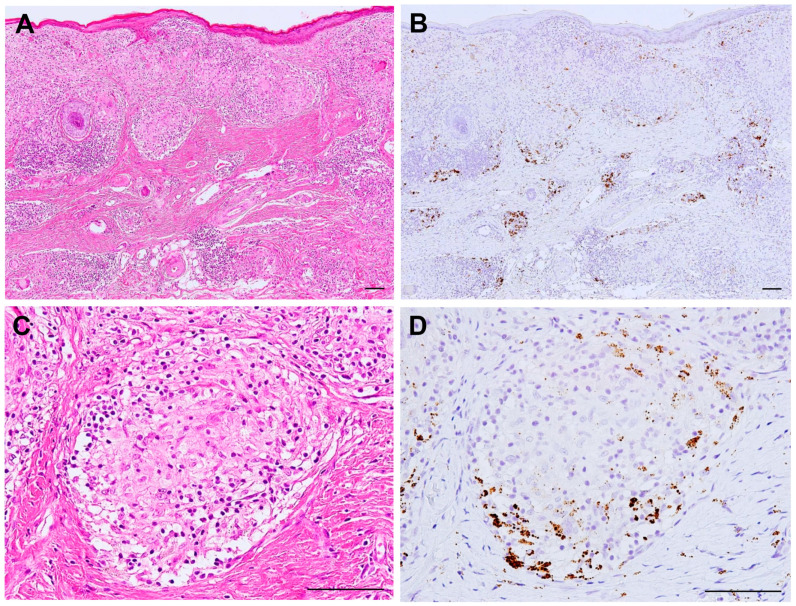
Active *P. acnes* infection in granulomatous inflammation of cutaneous sarcoidosis. Hematoxylin–eosin stain and immunohistochemistry with *P. acnes*-specific PAB antibody are shown pairwise. Many granulomas are found in the dermis with prominent lymphocytic infiltration (**A**). Many PAB-reactive *P. acnes* (in brown color) are found corresponding to the areas of granulomatous inflammation (**B**). In a non-caseating epithelioid cell granuloma (**C**), *P. acnes* is abundant in the peripheral area with more lymphocytic infiltration (**D**). All photos are original. Scale bar: 100 μm.

Histopathologic evidence of active *P. acnes* infection continues to accumulate (Figure 4). *P. acnes* induces interleukin-17 and IFN-ɣ production by T-cells [72], suggesting that mixed Th1/Th17 responses are promoted by this bacterium [73]. The association of *P. acnes* and autophagy is known [24] and the potential dysfunction of mTOR, Rac1, and autophagy-related pathways in sarcoidosis is suggested [74,75,76]. Notably, the host–commensal relationship relies on a unique Treg cell population that mediates tolerance to bacterial antigens during a defined developmental window [77]. Intravenous or intratracheal *P. acnes* inoculation causes self-limiting granulomatous inflammation in the liver [78,79] and lung [80,81], respectively. Interestingly, extrapulmonary sensitization to *P. acnes* induces pulmonary Th1 granulomas, primarily in subpleural and peribroncho-vascular regions, suggesting that commensal pulmonary *P. acnes* primes the host for the development of sarcoid-like pulmonary granulomatosis [82,83].

**Figure 5:**
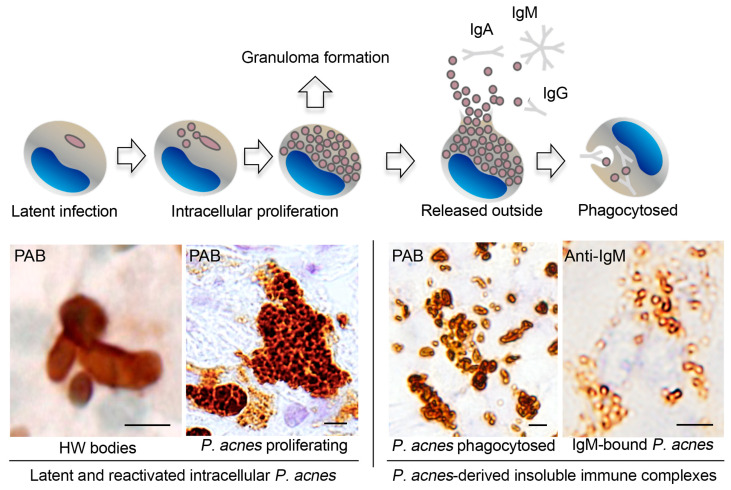
Intracellular proliferation of latent *P. acnes* followed by insoluble immune complex formation in sarcoid lymph nodes. Representative immunohistochemical features revealed by PAB or anti-IgM antibodies are shown below the illustrated macrophages with each stage of intracellular *P. acnes* manifestation. These findings imply that intracellular proliferation of latent *P. acnes* in paracortical macrophages triggers granuloma formation in sarcoidosis patients. PAB-reactive small round bodies bound with immunoglobulins (mainly IgM and IgA) are formed extracellularly and phagocytosed by sinus macrophages after release from paracortical macrophages with proliferating *P. acnes* that are free of immunoglobulins. Some of the *P. acnes*-derived immune complexes may escape local phagocytosis. All photos are original. Scale bar: 5 μm. HW, Hamazaki–Wesenberg.

According to the results of IHC with the PAB antibody, granulomatous inflammation in sarcoidosis patients may be caused by intracellular *P. acnes* proliferation at sites with latent infection (Figure 5). In sarcoid lymph nodes, many PAB-reactive *P. acnes* are bound with immunoglobulins in sinus macrophages, whereas those in paracortical granuloma cells are mostly free from immunoglobulins [84]. The difference between *P. acnes* with or without immunoglobulins suggests that *P. acnes*-derived insoluble immune complexes are formed extracellularly after intracellular proliferation and phagocytosed in sinus macrophages, some of which may escape local phagocytosis and spread via the lymphatic system and bloodstream.

**Figure 6:**
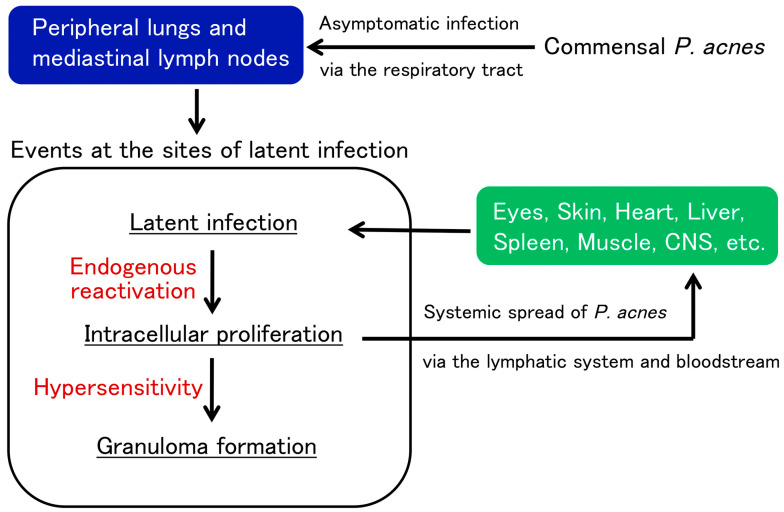
Potential pathogenesis of sarcoidosis as an allergic endogenous infection caused by *P. acnes*. Commensal *P. acnes* causes asymptomatic intracellular infection and persists in the peripheral lungs and mediastinal lymph nodes. Latent *P. acnes* is activated endogenously under certain conditions and proliferates intracellularly at the sites of latent infection. In patients with Th1 hypersensitivity to *P. acnes*, granulomatous inflammation is triggered by intracellular proliferation of the bacterium. Extracellular *P. acnes* that escaped granulomatous confinement or local phagocytosis potentially cause new latent infection in multiple organs via the lymphatic system and bloodstream dissemination. The latent infection spread in systemic organs can be simultaneously reactivated by additional triggering events, leading to granuloma formation at all sites of latent infection.

Translocation of *P. acnes* after local proliferation at the primary sites of latent infection (usually in the lung and mediastinal lymph nodes) may cause new latent infection (primarily in vascular endothelial cells) in systemic organs where later reactivation causes granuloma formation in predisposed individuals with Th1 hypersensitivity to the symbiotic bacterium (Figure 6) [49,50]. Alternatively, local entry and subsequent latent infection other than systemic spread of *P. acnes* may occur, such as in scar or tattoo sarcoidosis with a previous history of epidermal disruption and the salivary gland sarcoidosis with potential transductal epithelial infection.

Reactivation at the sites of latent infection may be triggered under certain host conditions, such as physical or mental stress, in sarcoidosis patients [85], as observed in patients with herpes zoster [86]. Tumor necrosis factor alpha inhibitors may cause tuberculosis or sarcoidosis by reactivating latent *M. tuberculosis* [87] or *P. acnes* [55] at sites with latent infection. Sarcoidosis is also induced by immune checkpoint inhibitors [88], which might be associated with disrupted peripheral T-cell tolerance against not only cancer cells, but also symbiotic *P. acnes*.

## 8. Conclusions

The results of many molecular and immunologic studies supporting mycobacterial and propionibacterial etiologies of sarcoidosis are complicated because of potential latent infection by both microorganisms in deep organs such as the lungs and lymph nodes. A mycobacterial etiology is based on hypersensitivity to some pathogenic antigens without active infection. A propionibacterial etiology is based on active *P. acnes* infection with the bacterium reliably detected in granulomas. *P. acnes* and *M. tuberculosis* are the only microorganisms with sufficient evidence for an etiologic role in sarcoidosis. More of the available histopathologic data support *P. acnes* as a cause of sarcoidosis compared with *M. tuberculosis*, suggesting that an endogenous infection by normally symbiotic *P. acnes* may cause granuloma formation in some predisposed individuals with Th1 hypersensitivity against intracellular proliferation of latent *P. acnes* triggered by certain host or drug-induced conditions. In the histopathologic diagnosis of sarcoidosis, *M. tuberculosis*-associated and *P. acnes*-associated sarcoidosis will possibly be differentiated in some patients by IHC for each etiologic diagnosis and potential antimicrobial intervention of sarcoidosis.

## Abbreviations

16S-rRNA: 16S-ribosomal ribonucleic acid; BAL, bronchoalveolar lavage; CI, confidence interval; DNA, deoxyribonucleic acid; ESAT-6, early secretory antigenic target-6; FFPE, formalin-fixed paraffin-embedded; HHV-8, human herpesvirus-8; HW, Hamazaki–Wesenberg; IFN-ɣ, interferon-gamma; IHC, immunohistochemistry; mKatG, mycobacterial catalase-peroxidase; MTC, *M. tuberculosis* complex; mTOR, mechanistic target of rapamycin; NOD1, nucleotide-binding oligomerization domain-containing protein 1; NTM, nontuberculous mycobacteria; OR, odds ratio; PCR, polymerase chain reaction; PPD, purified protein derivative; Rac1, Ras-related C3 botulinum toxin substrate 1.
